# Intracapsular Fractures at the Hip

**Published:** 1905-10-07

**Authors:** 


### INTRACAPSULAR FRACTURES AT THE HIP.

The diagnosis of intracapsular fracture of the neck of the femur is fully discussed in an article appearing in *International Clinics* (vol. ii., 1905) and written by the late Thomas H. Manley. With regard to the direction of the blow, he states that in more than 150 cases of intracapsular fractures coming under his own care in hospital and in private practice, in no single instance was the injury produced by force transmitted from the foot or knee. The force is always direct and applied over the great trochanter. In all but exceptional instances, when a person past middle age, and especially if the patient is an elderly female, has sustained a violent fall on the hip, with loss of function as an immediate result, it may be predicted, before examining the limb, that an intracapsular fracture has occurred.

The signs usually described as accompanying the injury are (1) crepitus, (2) shortening of the limb, (3) eversion of the foot, (4) flattening in the region of the trochanter. But it is well to bear in mind that any or all of these signs may be absent although fracture exists. Crepitus is frequently absent for several days, and violent manipulation to elicit it is to be deprecated whether an anæsthetic be used or not. The absence of osseous crepitus is the rule rather than the exception in this fracture. So it is with shortening of the leg. Primary shortening is not common. Besides which the lower limbs are often of slightly different lengths, and a difference of less than one inch cannot be accepted as indicating a fracture. To lessen this source of error the position of the upper border of the great trochanter should be observed. As time goes by the shortening becomes more and more marked. Eversion of the foot, in injuries about the hip, is of no value at all as a sign of fracture, because it is present in greater or less degree in all severe sprains of the hip.

Flattening in the region of the trochanter is often difficult to determine, especially in fat people, and on account of swelling. Later on, when the neck of the femur has become absorbed, as always happens, the flattening may be quite distinct.

From the foregoing it is evident that the diagnosis of intracapsular fracture is not easy until some time has elapsed since the injury. A mistake is of little consequence to the patient, since union of the bone is neither to be expected nor attempted, but it may be most disastrous to the reputation of his medical attendant. It is always good practice to hold a consultation with another practitioner in a doubtful case, so that the patient may be impressed with the difficulty and the responsibility for a mistake may be shared. A skiagram should also be obtained in every case in which circumstances render it possible.

